# Serine phosphorylation mimics of Aβ form distinct, non-cross-seeding fibril morphs†

**DOI:** 10.1039/d3sc06343g

**Published:** 2024-10-11

**Authors:** Kalyani Sanagavarapu, Georg Meisl, Veronica Lattanzi, Katja Bernfur, Birgitta Frohm, Ulf Olsson, Tuomas P. J. Knowles, Anders Malmendal, Sara Linse

**Affiliations:** a Biochemistry and Structural Biology, Department of Chemistry, Lund University Lund Sweden sara.linse@biochemistry.lu.se; b Yusuf Hamied Chemistry Department, University of Cambridge Lensfield Road Cambridge UK; c Cavendish Laboratory, Department of Physics, University of Cambridge JJ Thomson Avenue Cambridge UK; d Physical Chemistry, Department of Chemistry, Lund University Lund Sweden; e Department of Science and Environment, Roskilde University Roskilde Denmark

## Abstract

The self-assembly of amyloid-β peptide (Aβ) into fibrils and oligomers is linked to Alzheimer's disease (AD). Fibrillar aggregates in AD patient's brains contain several post-translational modifications, including phosphorylation at positions 8 and 26. These play a key role in modifying the aggregation propensity of Aβ, yet how they affect the mechanism of aggregation is only poorly understood. Here we elucidate the aggregation mechanism of Aβ42 peptides with phosphomimic mutations at these positions, with glutamine mimicking the size, and glutamate mimicking both the size and charge effect. We find that all variants are less aggregation-prone than wild-type Aβ42 with the glutamate mutants showing the largest reduction. Secondary nucleation is the dominant nucleation route for all variants, as confirmed using seeding experiments; however, its rate is reduced by about an order of magnitude or more for all variants relative to wild-type. S26Q and S26E fibrils fail to catalyse nucleation of wild-type monomers and *vice versa*, while the S8 variants co-aggregate more readily with wild-type. Ultrastructural analyses by cryo-electron microscopy and small angle X-ray scattering reveal an altered structure with longer node-to-node distance and smaller cross-section dimensions of S26Q fibrils. These results imply that structural compatibility between fibrils and monomer is a key determinant in secondary nucleation, and that small modifications can alter the preferred fibril structure, and thus its potential to induce aggregation of other variants. Overall, our results indicate that phosphorylation could play a key role in controlling aggregation propensity and may lead to the formation of distinct, non-cross-seeding fibril populations.

## Abstract 2

Left: One plane of an Aβ42 wt fibril with Ser8 and Ser26 marked. Right: Four aggregation time courses starting from a 1 : 1 mixture of Aβ42 wt and S26E, implying that the peptides fail to coaggregate and two separate types of fibrils are formed.

## Graphical abstract

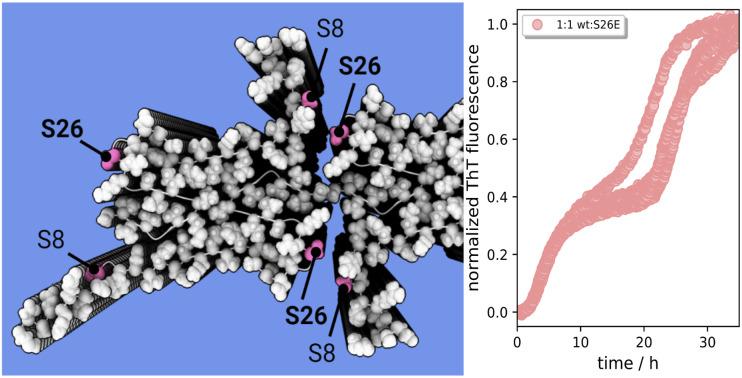

**Significance statement**

Phosphorylation of the amyloid β peptide is observed in the plaques isolated from brains of Alzheimer's disease patients. The effects on the amyloid β peptide aggregation rate and mechanism are investigated using mutations that mimic the size and charge effects of phosphorylation of serine residues 8 and 26. The results provide insights into position-dependent effects. While charge substitutions are retarding at both positions, the size substitution plays a role at position 26 only, leading to altered fibril structure and failure to cross-seed the wild-type peptide, implying a requirement for structural compatibility in surface-catalyzed nucleation.

## Introduction

Post-translation modifications (PTM) can have a pronounced impact on protein function and solubility and are observed with several proteins involved in neuro-degenerative diseases. For the amyloid β peptide (Aβ) associated with Alzheimer's disease (AD) a variety of PTM's have been observed in AD patients, including phosphorylation,^1–13^ N-terminal truncation and extension,^14–18^ oxidation,^19–22^ nitration,^23–26^ pyro-glutamate formation,^27–31^ glycosylation,^32–35^ racemization and isomerization,^28,36–38^ ubiquitination,^39–43^ SUMOylation,^44–49^ and covalent dimer formation.^18^

Phosphorylation increases the size and adds negative charge and additional hydrogen bonding functionality to the modified side-chains. Phosphorylation of a protein may therefore affect its folding, stability, and function, and can provide a recognition signal for the regulation of additional modifications. Phosphorylation is indeed one of the most commonly observed PTMs in the amyloid deposits in brains of AD patients.^2,3,7–11^ Some proteins, *e.g.* the microtubule associated tau protein, tend to become phosphorylated in the presence of Aβ,^50–52^ and earlier studies have provided evidence that phosphorylation of Aβ can affect protein–protein interactions, protein–lipid interactions and subcellular localization of amyloid proteins.^53–56^

**Fig. 1:**
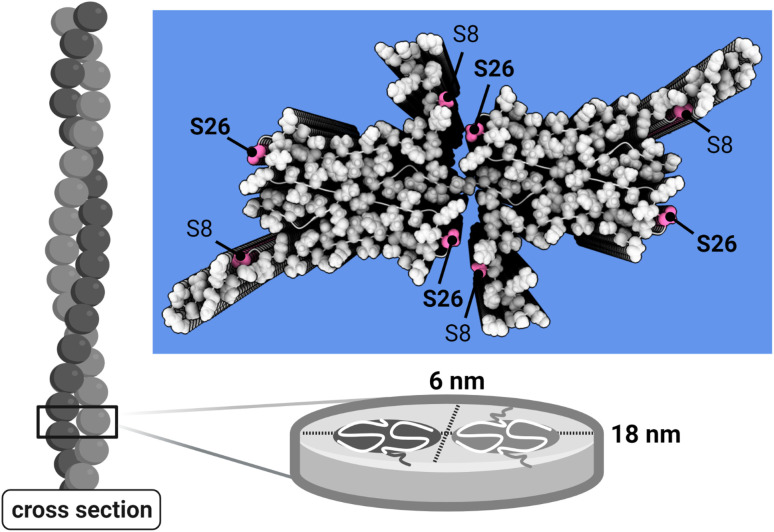
Aβ42 fibril model. Atomistic model of Aβ42 wt fibrils^58^ based on solid state NMR^59^ and SAXS data,^58^ with the serine residues in position 8 and 26 in pink, together with a schematic representation of fibril cross-section compatible with the SAXS data.

Phosphorylation requires a hydroxyl group and can thus occur on the side-chains of serine, tyrosine and threonine residues. The Aβ42 sequence contains one tyrosine residue at position 10 and two serine residues at position 8 and 26. In this study, we focused on phosphorylation of the serine residues because these are identified as phosphorylated *in vivo* in several reports.^2,3,7–11,13,57^ Ser8 is located in the flexible N-terminal part of Aβ42 while Ser26 is found on the surface of the ordered core of the fibrils and at the interface between the two filaments in a fibril (Fig. 1). Phosphorylation of Ser26 *in vivo* by phosphoinositide 3 kinase was found to reduce Aβ42 toxicity in *Drosophila* although the overall amount of amyloid deposits was unaltered.^11^*In vitro* experiments have indicated an overall retardation of Aβ40 aggregation upon S26E mutation.^60^

There are several methods to achieve protein phosphorylation *in vitro*. Specific protein kinases like serine or tyrosine kinases can be used to control phosphorylation of serine and tyrosine hydroxyl groups in the presence of ATP.^61–63^ Animal-derived or commercial kinase kits are available but may be an expensive route. Moreover, enzymatic phosphorylation is often promiscuous and difficult to control and may result in more or less random phosphorylation of the hydroxyl groups of a protein. To be specific, phosphorylation often needs to be optimized, and analytical methods such as SDS-PAGE and mass spectrometry are needed to confirm a specific phosphorylation pattern and an additional purification step is needed to remove the remainder of the compounds used for phosphorylation.

Another way to achieve *in vitro* phosphorylation includes synthesis of peptides with specific residues being phosphorylated.^7^ Although a highly controlled process, depending on their size, synthetic peptides may be contaminated by truncated variants of the same peptide, d-isomers of some residues, *etc.*

**Fig. 2:**
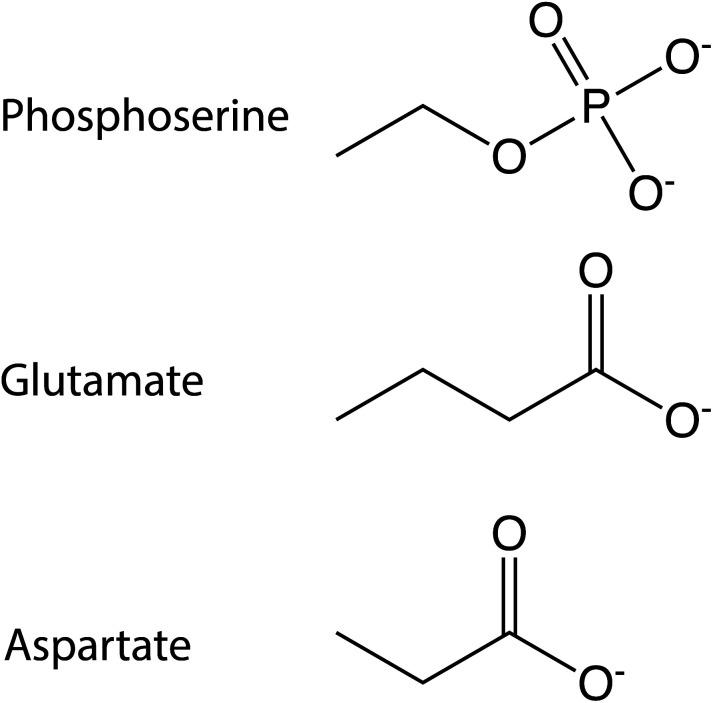
Chemical structures. The chemical structure of the side chains of phosphoserine, glutamate and aspartate residues.

A third approach, which avoids many of these drawbacks, involves the introduction of phosphomimic mutations of residues that could potentially undergo phosphorylation, offering control over the peptide homogeneity. Serine, tyrosine or threonine residues can be mutated to aspartate or glutamate^7,64,65^ to mimic the charge variation caused by a phosphate group. Mutation to glutamate may be more appropriate than aspartate because of a size more similar to the phosphate group (Fig. 2).

In the present work, we have studied the effect of such phosphomimic mutations on the aggregation process of Aβ42 using thioflavin T (ThT) fluorescence. Aβ42 was chosen for its higher toxicity and stronger connection to AD.^66,67^ We mutated serine residues 8 and 26 of Aβ42, one at a time, to glutamate to study the effect of increased side-chain volume and charge, and to glutamine to isolate the size effect. The aim was to understand the change in aggregation mechanism through analysis of the effects on the underlying microscopic steps. To this end we used global fitting of unseeded data. Self-seeding experiments with proteins of the same variant were performed to validate the nucleation mechanism and cross-seeding and co-aggregation experiments with different protein variants were used to understand the specificity of the mutational effects on secondary processes. The fibril morphology was studied using cryo-transmission electron microscopy (cryo-TEM) and small angle X-ray scattering (SAXS) to understand the striking lack of cross-seeding between wild-type (wt) and the Ser26 mutants.

## Results

### Aggregation of serine mutants into amyloid fibrils

**Fig. 3:**
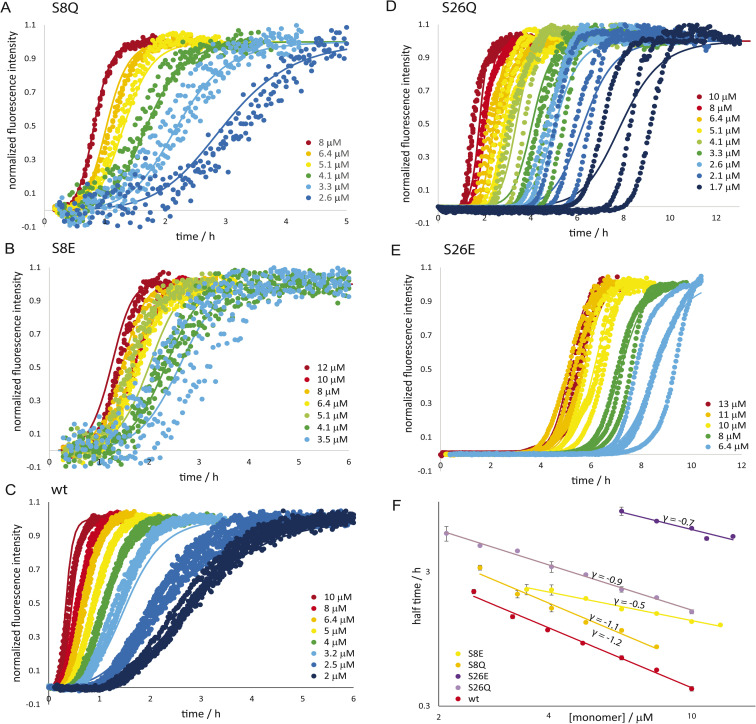
Aggregation kinetics. The aggregation kinetics for the phosphomimic mutants are shown as normalized ThT fluorescence *versus* time for a range of initial monomer concentrations of S8Q (A), S8E (B), wild-type wt; (C), S26Q (D) and S26E (E) in 20 mM sodium phosphate, 0.2 mM EDTA, 0.02% NaN_3_, pH 8.0, 37 °C. The half time of aggregation *versus* monomer concentration is shown for the five peptides are shown in panel (F).

The self-assembly into fibrils from supersaturated solutions of Aβ42 monomers with the serine mutations S8E, S8Q, S26E and S26Q was monitored by thioflavin T (ThT) fluorescence at 37 °C. This method relies on enhanced fluorescence intensity of ThT upon binding to β-sheet-rich fibrils. All the mutants were found to form fibrils in a time- and concentration-dependent manner, as evident by the sigmoidal-like curve shapes, but more slowly than the Aβ42 wt over the whole concentration range studied (Fig. 3A–E). The retarding effect is more pronounced for the variants with substitutions at position 26 compared to position 8. Furthermore, S26E is found to aggregate much more slowly than S26Q. This clearly illustrates that both the charge of peptide and the position of the substitution affect the rate of formation of new aggregates from monomers.

The half times of aggregation were extracted from each aggregation trace as the point in time where the ThT intensity is half-way between the initial baseline and the final plateau. This parameter provides a quantification of the overall aggregation propensity and is a convenient observable for exploring the monomer dependence of the aggregation reaction. The half times are plotted as a function of monomer concentration (Fig. 3F) and fitted by a power law function,

1 *t*_1/2_(*c*) = *Bc*^*γ*^

where *B* is a proportionality constant and *γ* is the scaling exponent for the monomer dependence of the half time, which provides mechanistic information about the reaction orders of the dominant nucleation processes.^68^ For example, *γ* would be −1.5 for a process fully dominated by secondary nucleation with a reaction order of 2, and close to −0.5 in cases of fully saturated secondary nucleation or a fragmentation-dominated process.^68,69^ Serine to glutamate mutants show a scaling exponent of reduced magnitude compared to wt (S8E *γ* = −0.5, S26E *γ* = −0.7, wt *γ* = −1.3, ref. 70), reflecting a reduced monomer concentration dependence. The scaling exponents for the serine to glutamine mutants are in between those of the glutamate mutants and wt (S8Q *γ* = −1.1, S26Q *γ* = −0.9).

### Global kinetic analysis

In order to determine the aggregation mechanism, we fit integrated rate laws derived from molecular mechanisms to the kinetic data in a global manner using the Amylofit interface.^69^ A multi-step secondary nucleation model was fitted globally to the aggregation kinetics data over the range of initial monomer concentrations. Assuming reaction orders are unchanged from the wt, this model has three variable parameters: the combined rate constant for elongation and primary nucleation (*k*_+_*k*_*n*_), the combined rate constant for elongation and secondary nucleation (*k*_+_*k*_2_) and the monomer concentration at half saturation of secondary nucleation √*K*_M_. The values of the fitted rate constant are shown in Table 1.

**Table 1:** Fitted rate constants

	Primary nucleation (*k*_*n*_ in M^−1^ s^−1^)	Elongation (*k*_p_ in M^−1^ s^−1^)	Secondary nucleation rate at 6.4 μM and 10% completion (M^−1^ s^−1^)	Secondary nucleus conversion (*k*_2_*K*_M_ in s^−1^)
wt	5 × 10^−6^	7 × 10^6^	4 × 10^−13^	8 × 10^−7^
S8Q	7 × 10^−5^	2 × 10^6^	6 × 10^−14^	2 × 10^−7^
S8E	2 × 10^−5^	4 × 10^6^	1 × 10^−14^	2 × 10^−8^
S26Q	3 × 10^−7^	1 × 10^6^	4 × 10^−14^	7 × 10^−8^
S26E	8 × 10^−11^	3 × 10^6^	4 × 10^−15^	1 × 10^−8^

**Fig. 4:**
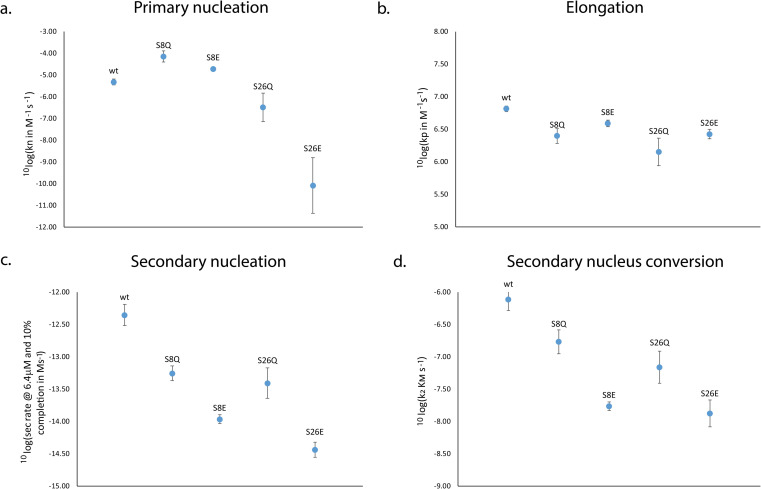
Rates and rate constants. Primary nucleation rate constant (a), elongation rate constant (b), secondary nucleation rate at 6.4 μM and 10% completion (c) and the rate of conversion of secondary nuclei (d) are shown for wt *vs.* the four serine mutants S8E, S8Q, S26E and S26Q. *Y*-axis values are on log scale.

S26E showed a significantly lower value of the combined parameter *k*_+_*k*_*n*_ compared to wt, while for the other mutants the values were similar to wt (Fig. 4A). Note that because primary nucleation is only a minor contributor to the kinetics, the errors associated with its rate constants determined from fitting are large. To deconvolute the effect on the rate constants for primary nucleation, *k*_*n*_ and elongation, *k*_+_, we estimated *k*_+_ using the fibril dimensions from TEM measurements (see methods). Through this approach, *k*_+_ was determined to be approximately within a factor of 3 of the wt for all mutants (Fig. 4B). Interpretation of differences in the rate constant of secondary nucleation is more difficult as the mutants may show a different degree of saturation. A more easily interpretable measure is the rate of secondary nucleation (rather than the rate constant), calculated at a specific peptide concentration. From the global fits, the rate of secondary nucleation was calculated at 6.4 μM peptide and 1% aggregation and was found to be approximately one order of magnitude lower than wt for the glutamine variants, and about two orders of magnitude lower than the wt for the glutamate variants (Fig. 4C). The concentration at half saturation of secondary nucleation, was found to be √*K*_M_ = 6 ± 2 μM for S8Q, 2.0 ± 0.4 μM for S26Q, and 6 ± 4 μM for S26E (Table 2). An upper bound value is given for S8E (√*K*_M_ < 3.5 μM) because secondary nucleation is fully saturated over the whole concentration range studied, thus we can only conclude that *K*_M_ lies below this range.

**Table 2:** √*K*_M_ (concentration where fibrils are half saturated with monomer, *n*_2_ = 2) values of wt and four serine mutants

	√*K*_M_ (μM)
wt	≥6 (ref. 71)
S8E	≤3.5
S8Q	6 ± 2
S26E	6 ± 4
S26Q	2 ± 0.4

The product of the values of *k*_2_ and *K*_M_ gives the maximal rate of secondary nucleus formation in analogy to the maximal rate in enzyme kinetics, *V*_max_.^71,72^ We find a reduction relative to wt for all mutants, close to the amount of reduction seen for the overall secondary nucleation rate (Fig. 3D). This indicates that the reduced secondary nucleation rate of the mutant proteins is predominantly due to a decreased conversion/detachment rate.

### Self-seeding

**Fig. 5:**
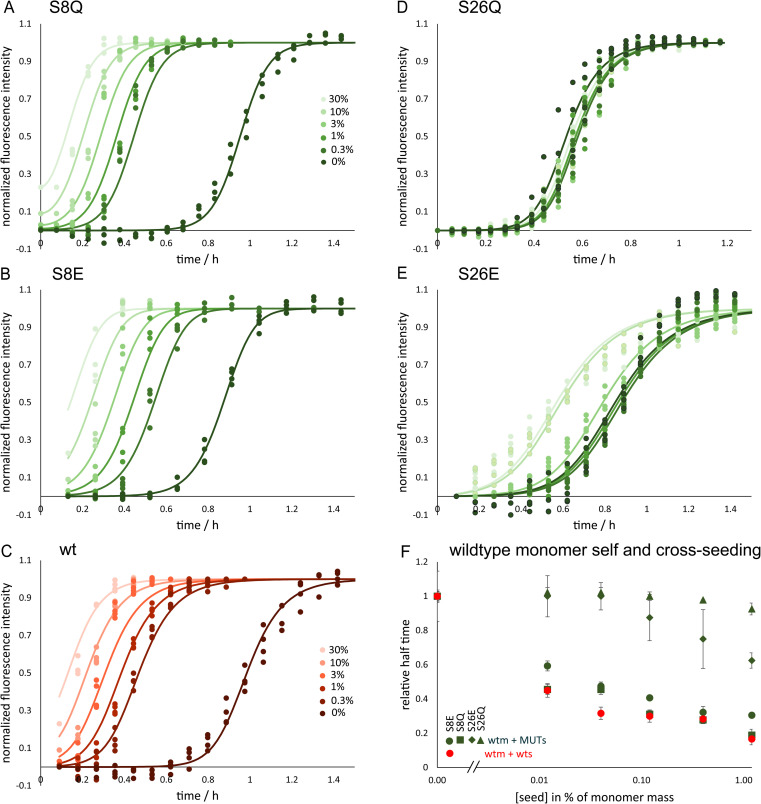
Self- and cross-seeding of wt monomers. Aggregation kinetics data are shown in the form of normalized ThT fluorescence intensity for reactions started for Aβ42 wt monomers without or with seeds of S8Q (A), S8E (B), wt (C), S26Q (D) and S26E (E) in 20 mM sodium phosphate, 0.2 mM EDTA, 0.02% NaN_3_, pH 8.0, 37 °C, with the color codes for seed concentrations in panel (A and C). The relative half time of aggregation for wt monomer (wtm) mixed with wt seed (wts, red) or mutant seeds (MUTs, green) is shown *versus* seed mass concentration in panel (F).

**Fig. 6:**
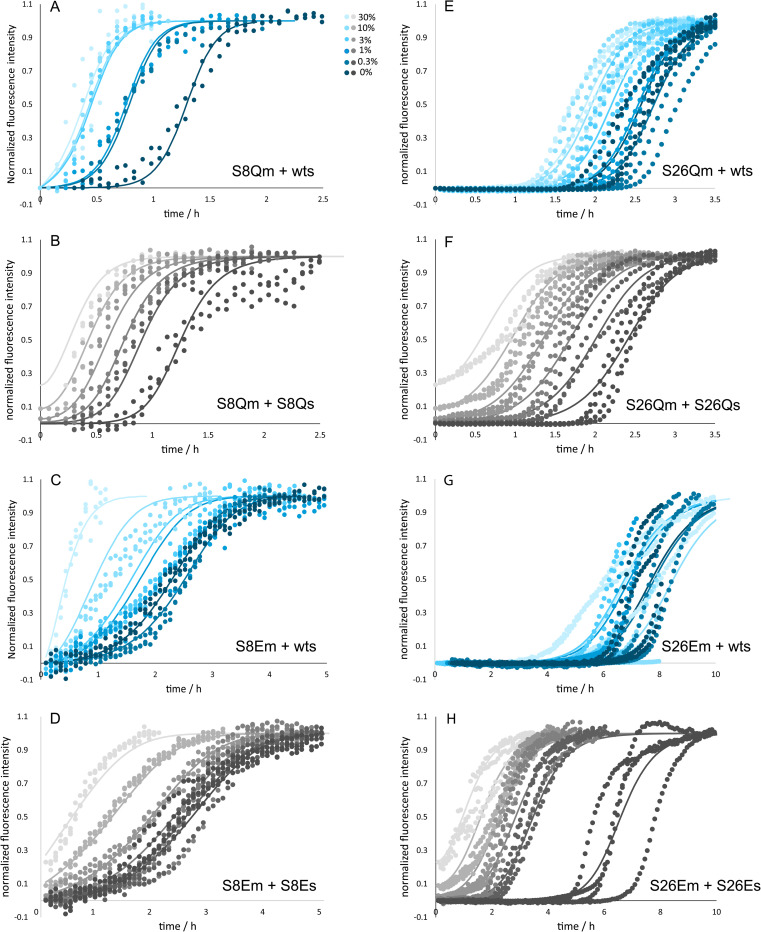
Self- and cross-seeding of mutant monomers. Aggregation kinetics data are shown in the form of normalized ThT fluorescence intensity for reactions started for mutant monomers without or with seeds of wt or the same mutant for S8Q (A and B), S8E (C and D), S26Q (E and F) and S26E (G and H) in 20 mM sodium phosphate, 0.2 mM EDTA, 0.02% NaN_3_, pH 8.0, 37 °C. The color codes for seed concentrations are shown in panel (A).

**Fig. 7:**
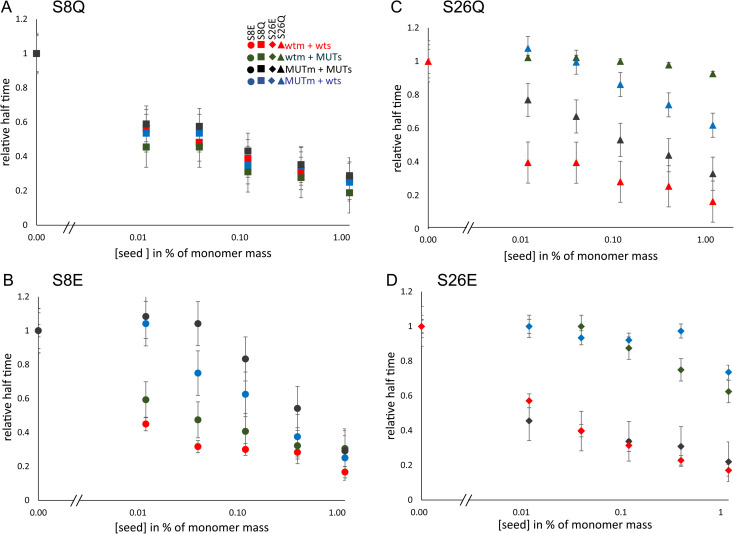
Self- and cross-seeding half times for mutant monomers. The half time of aggregation as a function of seed concentration for each mutant relative to the half time of non-seeded reactions (A–D). The color and symbol codes for seeds are shown in panel (A).

Self-seeding experiments, with preformed fibrils of the same protein variant added at the beginning of the reaction, were set up to validate the model that best described the unseeded data in global fitting. The results of self-seeding are displayed for Aβ42 wt in Fig. 5 and for the mutants in Fig. 6 and 7. In all cases the seed concentration is reported as monomer mass concentration in % of the free monomer concentration. All peptides studied show very efficient self-seeding, which is seen as a seed-concentration-dependent shortening of the lag phase. The significant reduction in aggregation half time at low seed concentration (0.3–3%) validates the finding of secondary nucleation being the dominant mechanism of new fibril formation in all cases.

### Cross-seeding

Cross-seeding experiments (seeds made from one protein to trigger the aggregation of monomer of another protein) were set up to investigate the residue specificity in surface-catalyzed nucleation (low seed concentration) as well as in elongation (high seed concentration). The cross-seeding of wt monomer with position 8 mutant fibril and of position 8 mutant monomers with wt fibril appear equally effective as the respective self-seeding cases (Fig. 5–7). However, for peptides with mutations at position 26, cross-seeding appears to be much less efficient than self-seeding (Fig. 5–7). The S26Q mutation was found to completely abolish the ability of fibrils to catalyse the aggregation of wt monomer at all seed concentrations studied. Likewise, the seeding of S26Q monomer with wt seed was highly inefficient, except for a slight acceleration observed at 30% seed (Fig. 6E). Also for S26E mutant monomer, the aggregation was accelerated by wt seeds at the highest seed concentration only (30%). In the case of wt monomer cross-seeded with S26E mutant seed, half times were not affected by 0.3 or 1% seed and reduced only at 3–30% seed. The results of cross-seeding in the low seed regime are summarized in Table 3.

**Table 3:** Self and cross-seeding in the low seed regime. √ = effective seeding. X = no seeding. N/A = not applicable; these fields are the same as self-seeding

	Self-seeded	Cross-seeded by wt	Cross-seeds wt
wt	√	N/A	N/A
S8Q	√	√	√
S8E	√	√	√
S26Q	√	X	X
S26E	√	X	X

The cross-seeding of the mutants was further evaluated using the rate constants obtained from the unseeded aggregation of monomeric peptides. The data for peptides with mutations at position 8 are well fitted under the assumption that surface catalysis of monomer of one species on the seeds of other species is as effective as self-seeding (Fig. 6). The very weak cross-seeding effect between wt and peptide mutated at position 26 precluded such analysis.

### Co-aggregation

**Fig. 8:**
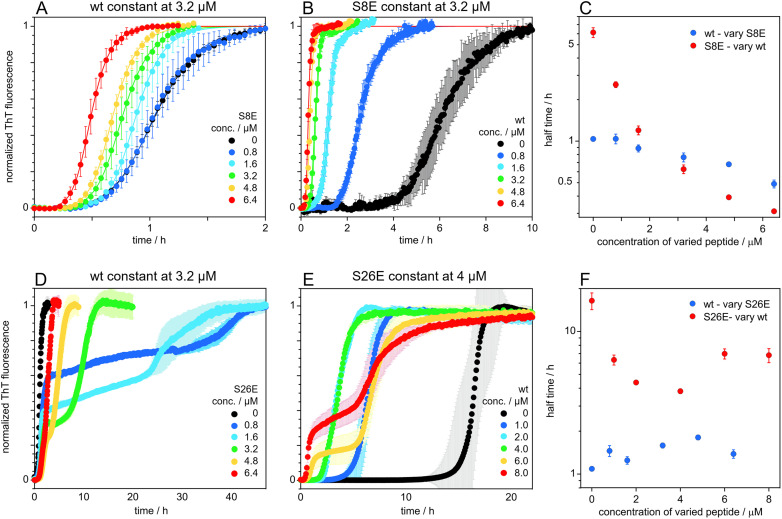
Co-aggregation data. Co-aggregation kinetics starting from monomer mixtures with either constant concentration of wt and that of mutant varied (A and D) or constant concentration of mutant and that of wt varied (B and E) in 20 mM sodium phosphate, 0.2 mM EDTA, 0.02% NaN_3_, pH 8.0, 37 °C. All data are shown as normalized ThT fluorescence intensity with y-average and standard deviation over 4 replicates. Data with S8E are shown as in panels (A and B), with extracted half-times in panel (C). Data with S26E are shown in panels (D and E), with extracted half-times in panel (F). In panel (E), the data for S26E alone was both *x*- and *y*-averaged, with the standard deviations calculated for *y*-averaging only. The blue data points in panel (F) are the half times for the first transition when wt is constant, and the red data are the half times for the second transition when S26E is constant.

The S8E and S26E mutants were evaluated in coaggregation experiments starting from mixtures of wt and mutant monomers monitored by ThT fluorescence. Such co-aggregation experiments can give insights into the compatibility of different proteins and their ability to form mixed aggregates. For incompatible proteins, that form aggregates consisting mostly of one type of protein, one can see characteristic double sigmoidal aggregation curves, with each increase in signal corresponding to the formation of a different type of fibril, as previously observed for mixtures of Aβ42 and Aβ40.^73^ In a first set of experiments, the initial monomer concentration of wt was held constant at 3.2 μM and that of mutant (S8E or S26E) was varied over 0, 0.8, 1.6, 3.2, 4.8 and 6.4 μM (Fig. 8A and C). In a second set, the mutant (S8E or S26E) monomer concentration was constant and the wt concentration was varied (Fig. 8B and D). A single transition is observed for each peptide alone, and for all mixtures of wt and S8E (Fig. 10A and B). For both wt and S8E, the half time of the single transition occurs earlier the higher the overall concentration of peptide (Fig. 8C). In contrast, the data for experiments starting from monomer mixtures of wt and S26E display two transitions (Fig. 8D and E). The half-time of the first transition is relatively constant, with a slight delay in the presence of S26E (Fig. 8F). S26E alone at 4 μM shows a single transition, while the data at 4, 6 and 8 μM wt display two transitions and for each transition, the half-time is shorter the higher the wt concentration (Fig. 8F). In another set of experiments, the total initial monomer concentration was held constant, and the ratio of mutant : wt varied (Fig. S2†). Again, the position 8 and position 26 mutants produce clearly different data. A single sigmoidal transition is observed for all S8E : wt mixtures whereas several of the S26E : wt mixtures produce data with two consecutive sigmoidal transitions (Fig. S2†).

### Ultrastructure of aggregates

**Fig. 9:**
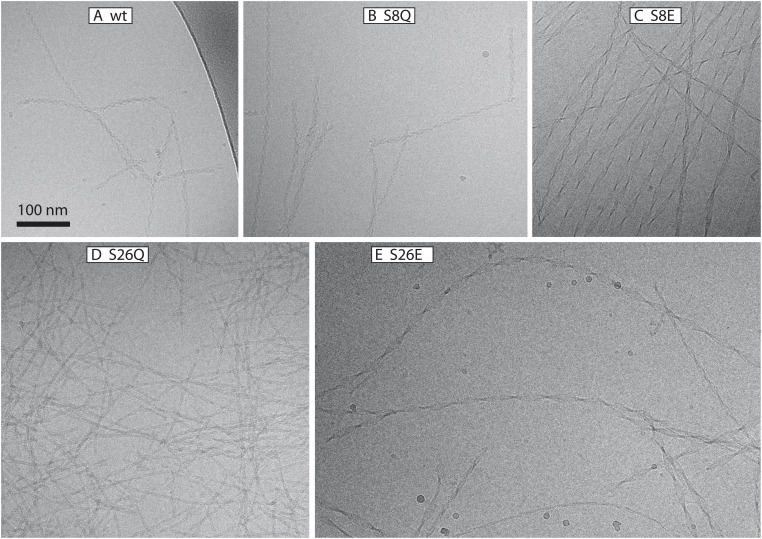
Cryo-TEM. Cryo-TEM images of Aβ42 wt (A) and serine phosphomimic mutant (B–E) fibrils freshly formed from samples of 10 μM monomer in 20 mM sodium phosphate, 0.2 mM EDTA, 0.02% NaN_3_, pH 8.0, 37 °C.

The cryo-TEM images reveal that Aβ42 wt and all variants form fibrils with at least two filaments winding around each other with a defined twist distance (Table 4). Fibrils of S8Q are very similar to wt fibrils in terms of the overall fibril length and the relatively tight twist, *i.e.* short node-to-node distance. The images further indicate that the fibrils of S8E, S26E and S26Q are longer than the wt fibrils. Wider fibrils and significantly longer twist distances are observed for the more negatively charged variants S8E and S26E (Table 4), while the width of the S26Q fibrils appears to be smaller than for wt fibrils (Fig. 9).

**Table 4:** Node to node distance of Aβ42 wt and serine phosphomimic mutant fibrils as observed from cryo-TEM images (Fig. 9). Each average and standard deviation is based on measurements on 320 fibrils

	Node-to-node distance (nm)
wt	16.5 ± 5.3
S8E	57.6 ± 5.6
S8Q	17.0 ± 5.9
S26E	59.1 ± 5.7
S26Q	17.3 ± 8.8

### Fibril cross section

**Fig. 10:**
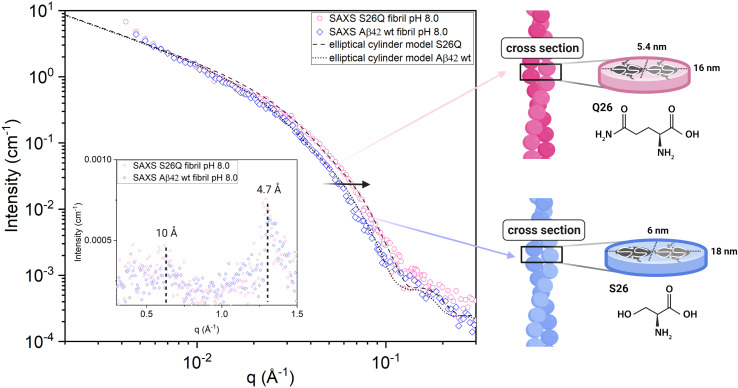
SAXS data. Small angle X-ray scattering patterns of Aβ42 S26Q fibrils (pink, four independent replicates are presented) and Aβ42 wt fibrils (blue, data taken from ref. 70) formed in 20 mM sodium phosphate, 0.2 mM EDTA, 0.02% NaN_3_, pH 8.0. The dotted and broken lines are calculated scattering patterns, where the fibrils are modeled as elliptical cylinders. Schematic representations of S26Q (pink) and wt (blue) fibrils and the respective fibril cross-sections that fit the SAXS data are shown to the right of the scattering data.

Small- and wide-angle X-ray scattering (SAXS and WAXS) was used to investigate in more detail the number of filaments and dimensions of the cross section of fibrils formed from Aβ42 S26Q in solution, using the same buffer conditions as in the kinetics experiments. In Fig. 10 we compare the SAXS patterns obtained from Aβ42 S26Q and wt Aβ42 fibrils, plotted as intensity, *I*(*q*), *versus* the scattering vector, *q*. In both samples the protein concentration was 350 μM. The SAXS data were analyzed using an elliptical cylinder model (Table 5 and Fig. 10), as described in detail in ref. 58. In short, the model scattering intensity is given by2
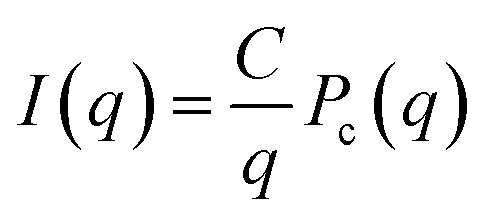
where *P*_c_(*q*) is the normalized fibril cross-section form factor, and *C* is given by3
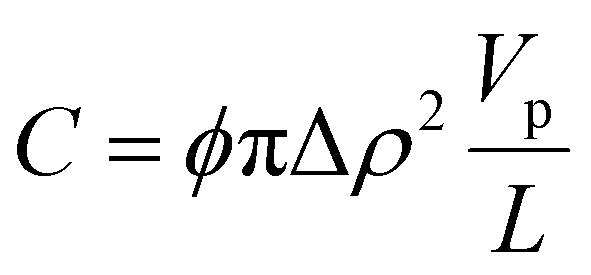
here, *ϕ* is the fibril (protein) volume fraction, Δ*ρ* = *ρ*_p_ − *ρ*_b_ is the difference in scattering length density between protein (p) and buffer (b), and *V*_p_/*L* is the protein volume per unit length in the fibrils. The protein volume fraction, *ϕ*, is related to the molar concentration, *c*, as *ϕ* = *cM*/*d*_m_, where *M* is the molar mass and *d*_m_ is the protein mass density. Here we assume *d*_m_ = 1.43 g cm^−3^.^74^ The fibrils can be described as composed of stacks of essentially two dimensionally folded protein molecules, where each stack contains a number of parallel intermolecular β-sheets propagating in the fibril direction, with a stacking periodicity of *d*_β_ = 4.7 Å. Two parallel (intertwined) stacks are considered to make up one filament.^58^*V*_p_/*L* reports on the number of filaments, *N*, in the fibrils as we have

4 *V*_p_/*L* = 2*Nv*_p_/*d*_β_

where *v*_p_ = 5.4 nm^3^ is the Aβ42 molecular volume. Finally, the normalized cross-section form factor of an elliptical cylinder can be written as5
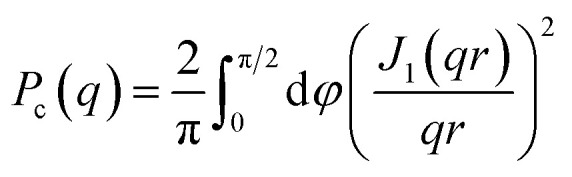
with *J*_1_(*x*) being the first order Bessel function and *r* = ((*a* sin *φ*)^2^ + (*b* cos *φ*)^2^)^1/2^ is an apparent cross-section radius that varies with the polar angle *φ*. Based on eqn (2)–(5) we have calculated scattering curves, adjusting parameters and compared with the experimental data. Shown as solid lines in Fig. 10 are calculated scattering curves that describe reasonably well with the data. Model parameters are summarized in Table 5. The results show that Aβ42 S26Q fibrils have a similar but slightly smaller cross-section (semi-axes 2.7 and 8 nm) compared to Aβ42 wt fibrils (semi-axes 3 and 9 nm; ref. 58). At lower *q*-values, where *P*_c_(*q*) approach unity, both samples show the same scattered intensity, *I*(*q*) = *C*/*q*, with the same *C* value. From the *C* value we obtain *N* = 2, hence both fibrils consist of two filaments. The WAXS peak at *q* = 1.3 Å^−1^, associated with the periodic 4.7 Å distance between β-strands, is the same for the mutant and wt fibrils.

**Table 5:** SAXS elliptical cylinder model fit parameters for Aβ42 fibril samples formed in 20 mM sodium phosphate, 0.2 mM EDTA, 0.02% NaN_3,_ pH 8.0, at 37 °C

Model parameters, pH 8.0	wt fibril	S26Q fibril
Protein concentration [μM]	350	350
Molecular weight [g mol^−1^]	4645	4684
Protein mass density [g mL^−1^]	1.43	1.43
Buffer (solvent) scattering length density [cm^−2^]	9.47 × 10^10^	9.47 × 10^10^
Protein scattering length density [cm^−2^]	12.7 × 10^10^	12.7 × 10^10^
β-sheet repeat distance [Å]	4.7	4.7
Bg: Background [cm^−2^]	2.0 × 10^−4^	2.0 × 10^−4^
*a*: Semi-axis 1 [nm]	3	2.7
*b*: Semi-axis 2 [nm]	9	8.0
*N*: number of filaments	2	2

### A11-reactive intermediates

Dot blots were used to monitor the time-evolution of intermediates binding to the A11 primary antibody. As shown in Fig. S4,† Aβ42 wt and Aβ42 S26E display a similar amount of A11-reactive intermediates and a similar time evolution with respect to *t*_1/2_.

## Discussion

Several studies have identified phosphorylated Aβ peptides in body fluids and in the deposits of AD patient brains and highlight the importance of *in vivo* phosphorylation for the stability of fibrils and for oligomer formation.^1–12^ To assess the effect on the Aβ self-assembly rate and mechanism of phosphorylation at specific sites, the current study uses phosphomimic Aβ42 peptide mutants with size and charge modifications at positions 8 and 26, which are commonly observed to be phosphorylated *in vivo*. An advantage of mutagenesis is the generation of 100% modified peptide for conclusive biophysical studies. A limitation of the Ser to Glu or Gln mutations is their inability to fully mimic the size and hydrogen bonding capacity of a phosphoryl group.

In particular, we evaluate the influence of the phosphomimic modifications on the toxic oligomer producing secondary nucleation step. This step has many similarities to enzyme-catalysed reactions, in which substrate binding is followed by product formation and release, and in which the rate of product formation saturates at high substrate concentrations.^71,72^ Secondary nucleation occurs on the surface of fibrils, which catalyze nucleation. The process may involve the attachment of monomers to the fibril surface and detachment/conversion of the new aggregates or oligomers that can then further elongate and form mature fibrils. The fibril surface may be saturated by bound species and, with a reaction order of 2, the concentration at which the process is half saturated is given by √*K*_M_. At high monomer concentration, the rate of secondary nucleation thus becomes independent of monomer concentration and is instead dependent only on the rate of conversion/detachment of new aggregates from the fibril surface. The conversion/detachment process is modelled as independent of the monomer concentration. Thus, at high monomer concentration the detachment/conversion process becomes rate-limiting and the product *k*_2_*K*_M_ gives the rate of detachment/conversion, or the maximal rate of nucleus production, *V*_max_, in analogy to enzyme kinetics. As a general guideline: a decrease in the reaction order (*i.e.* the scaling exponent decreases in magnitude) indicates a shift to a more saturated system. In combination with consideration about the change in overall speed of the reaction, one can then infer how the individual processes of detachment/conversion and attachment are affected by the mutation.

In the following we first discuss each mutated position and the results obtained for each mutant from non-seeded and self-seeded aggregation kinetics, and structural investigations. After that we discuss the results of co-aggregation and cross-seeding studies and connect this to the mechanism of secondary nucleation on fibril surfaces.

### Position 8

A change in size at position 8 has little effect on the aggregation mechanism of the Aβ42 peptide and the fibrils of S8Q appear similar to those of wt Aβ42. There is limited change in the microscopic rate constants, and the degree of saturation of secondary nucleation for the S8Q mutant relative to wt Aβ42. Both the monomer binding affinity and conversion/release rate of secondary nucleation are somewhat reduced compared to wt, while the primary nucleation rate is slightly increased (Fig. 4D). Self and cross-seeding with wt are equally effective and the morphology of S8Q fibrils is also similar to that of wt fibrils. The addition of negative charge at position 8, as in S8E, mimicking both the change in size and charge upon phosphorylation of Aβ42, keeps the peptide fully compatible with wt in both cross-seeding directions and co-aggregation experiments reveal full mixing of the Aβ42 wt and Aβ42 S8E with a single transition observed at all peptide concentrations and ratios.

A significant effect is seen for the S8E self-aggregation rate relative to wt. The aggregation of S8E is slower than for the wt, mainly due to a change in the microscopic rate of secondary nucleation conversion/detachment by about one order of magnitude. Our results thus imply that an increased magnitude of the negative charge of Aβ42 interferes with secondary nucleation. The effect can most likely be ascribed to long-range electrostatic repulsion between monomers and between monomers and the fibril surface, which slow down this auto-catalytic step. This has been shown to be a general phenomenon.^75–79^ The fibrils of S8E are longer than those of the wt, in line with the lower rate of secondary nucleation but an unchanged elongation rate, meaning that fewer new fibrils are formed for every elongation event, leading to fewer and longer fibrils. The wt peptide seems to from co-aggregates with the position 8 phosphomimic mutant S8E and there seems to be full compatibility on all levels including primary and secondary nucleation as well as elongation (Fig. 8A and B).

### Position 26

S26Q is mimicking the change in size due to phosphorylation. The overall aggregation of S26Q is slower than for the wt. The data are described well by the multi-step secondary nucleation model. The microscopic rate constants for elongation and secondary nucleation are slightly lower than for the wt. The saturation concentration √*K*_M_ of S26Q is 2 ± 0.4 μM, is lower than for the wt. The detachment/conversion rate constant of new aggregates is also lower (7 × 10^−8^ s^−1^) than for wt (8 × 10^−7^ s^−1^). The self-seeding data support the presence of a secondary nucleation process as predicted based on the non-seeded data. Based on cryo-TEM and SAXS data we find a loose network of S26Q fibrils with tight twists and a slightly smaller cross-sectional area than wt fibrils.

S26E is mimicking both the change in size and charge due to phosphorylation. For the shorter alloform, Aβ40, an earlier study found complete loss of fibril formation upon phosphorylation of Ser26.^60^ The current results show that the aggregation of Aβ42 S26E is slower than for Aβ42 wt and is fitted well with a multi-step secondary nucleation model. The microscopic rate constant of secondary nucleation is lower than for the wt. The value of the saturation concentration √*K*_M_ for S26E (6 ± 4 μM) cannot be distinguished from wt. The product *k*_2_*K*_M_ is estimated to be 1 × 10^−8^ s^−1^ for S26E, which is lower than for wt.

Hence for both S26Q and S26E, the rate of conversion/detachment of new aggregates is reduced determining the overall lower aggregation rate (Fig. 4D). The reduction in oligomer conversion rather than oligomer generation is corroborated by the dot blot data, showing similar amounts of A11-reactive intermediates for Aβ42 wt and S26E (Fig. S4†). A11 has been reported to interact more strongly with the more hydrophobic oligomers than with fibrils.^80–86^ The self-seeding data support the predicted model and imply efficient secondary nucleation in both cases. For S26E, with strongly reduced rate constant for primary nucleation, this mean that secondary nucleation is even more dominant compared to wt, whereas for S26Q the dominance of secondary nucleation is as high as for wt Aβ42. The results of the co-aggregation experiments starting from monomer mixtures (Fig. 8C and D) imply that the wt peptide and the position 26 phosphomimic mutant S26E segregate into homomolecular fibrils, although there are some interactions that accelerate the nucleation of the slower S26E peptide, in a similar manner as observed for mixtures Aβ40 and Aβ42.^73^

### Seed specificity in surface catalysed nucleation

The results of the cross-seeding experiments provide insights to the specificity of the molecular mechanisms of Aβ fibril formation. While no specificity was detected for the position 8 mutants, the cross-seeding data points to a remarkable residue specificity at position 26 in terms of fibril-catalyzed nucleation. It is very clear that cross-seeding of S26E or S26Q monomer with wt fibrils is weak. For example, the cross-seeding of wt monomer by S26E or S26Q seed is totally abolished (Fig. 5). The cross-seeding data imply a complete failure of S26Q fibrils to catalyse the nucleation of wt monomers, even at high seed concentrations.

Specific catalytic sites for secondary nucleation may occur at well-defined locations of the fibril.^83^ Analogous to enzyme catalytic sites, secondary nucleation may require specific catalytic sites on the fibril surface for efficient catalysis. A recent study showed that the catalytic sites are relatively sparse along the fibril surface, most likely at defects.^84^ While the wt seeds (*i.e.* with Ser26) are highly effective in promoting the surface catalysed nucleation of wt monomers, mutant seeds with Glu26 or Gln26 seem to lack this capability. However, the fact that each of S26Q and S26E display a self-assembly mechanism dominated by secondary nucleation means that each mutant forms fibrils with a catalytic surface. The lack of cross-catalysis of the amyloid formation of wt peptide thus must have another origin than simply a general lack of catalytic surfaces.

The difference in cross-seeding *versus* self-seeding is likely related to an altered fibril structure of the position 26 variants, in line with our earlier observation for other Aβ42 mutants.^85^ In the model for wt fibrils based on SAXS, cryo-TEM and ssNMR data (Fig. 1) the Ser26 side-chains of two of four monomers per fibril plane are found at the interface between the two filaments (ref. 58; Fig. 1). Accommodation of the bulkier glutamine side-chain at these sites is likely impossible, which would explain the variant fold of the mutant fibril. A similar situation would arise with glutamate in position 26 and indeed the fibrils of S26E are observed to be longer and more loosely twisted than those of wt Aβ42. The failure of S26E fibrils to catalyse the nucleation of wt peptide would then also be reconciled with an altered fibril fold.

A change in structure may alter the surface properties and thereby interfere with nucleation on the fibril surface. Additionally, the fibrils may have a templating role along their surface or at exposed monomer planes at the sparse defects^84^ and only monomers that can take up the structure of the seed fibril may be able to nucleate on their surface, as was previously concluded for hydrophobic surface mutants of Aβ42.^85^ Whether such templating is related to growth along the existing fibrils prior to detachment, or due to nucleation at defects with exposed fibril interior, remains to be established. However, it is likely related to the variant fibril structure being unstable with a serine residue at position 26 and therefore it cannot be formed by wt. Likewise, the wt fibril structure would be unstable with the more bulky glutamine residue occupying position 26 and therefore it cannot form from S26Q peptide.

The lack of cross-seeding of wt monomer by S26Q seeds or S26E seeds can be explained by a simple assumption that in their presence, wt monomer can only undergo homogeneous secondary nucleation and elongation as happens in the absence of mutant seeds (Fig. 5). On the other hand, cross-seeding of mutant monomers on wt seeds, albeit much less effective than self-seeding, can be explained by non-specific heterogeneous nucleation of the mutant on wt seeds.

### Cross-seeding competent and non-competent variants

**Fig. 11:**
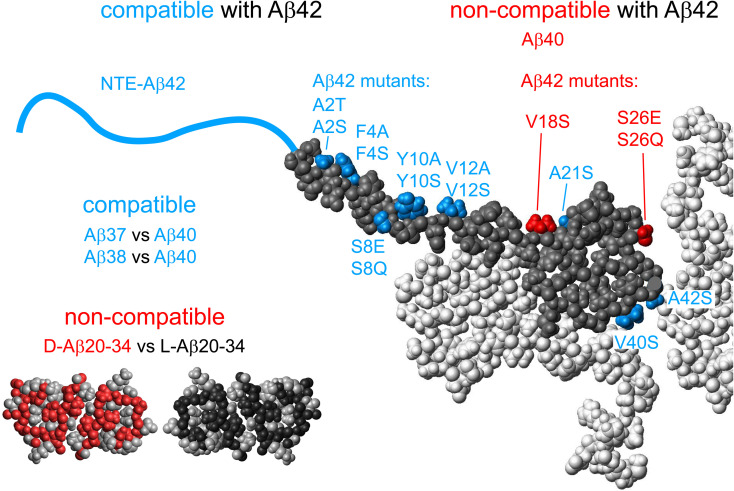
Cross-seeding competence. Summary of peptides that are compatible (blue) or non-compatible (red) in the nucleation on the fibril surface of each other. Single point mutations or other variants of the Aβ42 peptide, which allow cross-seeding with Aβ42 wt are marked in blue on the grey monomer unit from the structure of Aβ42 fibrils.^58^ Those that do not allow cross-seeding are marked in red. The other three monomer units in the fibril plane are shown in white. The non-compatible stereoisomers of Aβ20-34 are illustrated by the 6OIZ.pdb structure^87^ with the chiral back-bone and the two Ile side-chains in black and non-chiral side-chains in grey and its mirror image in red (chiral units) and grey (non-chiral).

The contrasting behavior of position 8 and position 26 mutants in terms of cross-seeding with wt, can be viewed in the light of other findings of cross-seeding competent and non-competent variants. As shown in Fig. 11, the cross-seeding competent Aβ42 variants include N-terminal extensions ranging from 5 to 40 residues, which seem to form fibrils with the same core structure as wt, and are equally potent in self- and cross-seeding with wt both in the low seed secondary nucleation regime and in the high seed elongation regime.^15^ Other examples in this category are hydrophobic surface mutations A2T, A2S, F4A, F4S, Y10A, Y10S, V12A, V12S, A21S, V40S and A42S.^85,86^ In addition, Aβ40 fibrils seem fully seeding competent *versus* Aβ37 and Aβ38 monomers, and *vice versa*.^88^ The non-competent variants form clearly distinct fibril structures that fail to cross-seeding with wt. Striking examples are Aβ42 *versus* Aβ40 in the form of sequence homogeneous recombinant peptides,^73^ peptides of identical sequence but opposite chirality forming mirror image fibril structures non-competent in seeding of the opposite stereoisomer,^89^ and Aβ42wt *versus* variants containing the V18S substitution.^85^ For α-synuclein, fibrils formed under one solution condition fail to propagate that structure through secondary nucleation under conditions where another fold is more stable.^90^ All these examples are compatible with a templating role of secondary nucleation, making it highly efficient only under conditions where the incoming monomers can form a stable aggregate with the same fold as of the parent fibril. If secondary nucleation indeed happens at rare defects,^84^ with exposed monomer planes, one could image that templating involves the formation of planes of identical fold, akin to elongation, but likely as single filaments or protofilaments.^91^ Because this occurs at a defect, it does not extend the mother fibril but rather after some layers detaches as an offspring fibril, *i.e.* the fibril number concentration increases through secondary nucleation.

## Experimental

### Purification and expression of mutant peptides

The mutant peptides were expressed from synthetic genes in fusion with the self-cleaving EDDIE tag^92^ cloned into the Pet3a vector (purchased from Genscript, Piscataway, New Jersey). The plasmid was transformed into *E. coli* to facilitate over-expression of the fused peptide in inclusion bodies. Overnight expression of each mutant peptide in auto-induction medium was performed as described previously.^93^

Cell pellet from 1.5 L culture was sonicated 5 times in 10 mM Tris/HCl, 1 mM EDTA, 1 mM DTT pH 8.5 (buffer A) with a trace of DNase, 50 mL each time. Each sonication step was followed by a centrifugation for 7 min at 15 000 rpm and the pellet was collected.

The inclusion body pelleted after the 5th sonication was dissolved in 70 mL 10 M urea in buffer A by sonication and the solution was then diluted with 80 mL buffer A and loaded onto a 20 mL DEAE-FF column pre-equilibrated with 4 M urea in buffer A. After loading, the column was washed with 100 mL 4 M urea in buffer A and eluted by a linear gradient from 0–0.4 M NaCl in buffer A with 4 M urea, total gradient volume 150 mL, flow rate 1 mL min^−1^. Eluted fractions were analysed by SDS PAGE and pools were made depending on how pure the fractions were. Each pool was diluted 15 times with 1 M Tris, 1 mM EDTA, 5 mM DTT, pH 7.8 in a glass bottle, an incubated at 4 °C for 48–72 h. EDDIE cleavage was monitored by SDS PAGE analysis (Fig. S1†). The cleaved sample was dialyzed over night against 10 mM Tris in cold room to reduce the Tris concentration to enable a second round of IEX on 50 mL of Q-sepharose big beads. Before use, the resin was washed on a Büchner funnel with 200 mL water, 200 mL 10 mM Tris/HCl pH 8.5 (buffer B) with 1 M NaCl, 4 × 100 mL of buffer B and then drained. The cleaved mutant peptide solution (2–4 L) was added to the conditioned resin and stirred now and then during one hour. The resin was collected on a Büchner funnel and the flow through checked with SDS PAGE (Fig. S1†). The resin was washed with 4 × 100 mL of buffer B, followed by 100 mL of buffer B with 10 mM NaCl. All washes were collected and checked with SDS PAGE (Fig. S1†). The cleaved mutant peptide was eluted with 4 × 80 mL buffer B, each with 75 mM NaCl and 100 mM NaCl.

All fractions that contain mutant peptide were lyophilized. Dried samples were dissolved in 6 M GuHCl for SEC on a 26 × 600 mm Superdex75 column in 20 mM sodium phosphate, 0.2 mM EDTA, pH 8.0. Collected fractions from the main peak were pooled and lyophilized. Dried samples were purified further with another SEC on the 26 × 600 mm Superdex75 column in 20 mM sodium phosphate, 0.2 mM EDTA, pH 8.0. All fractions were analysed on SDS-PAGE. The peak fractions were pooled and split into multiple identical aliquots, lyophilized and SEC on a 10 × 300 mm Superdex75 column was done just before setting up aggregation kinetics. MALDI MS for intact weight and ESI-MS after tryptic digestion confirmed the correct peptide sequence (Fig. S3†).

### *In vitro* aggregation kinetics of selected mutants

The freshly prepared monomer solution in 20 mM sodium phosphate buffer with 0.2 mM EDTA pH 8.0, as well as the same buffer without peptide, were supplemented with 6 μM ThT (from CalBiochem, prepared as a 2 mM stock, filtered through 200 nm filter) and kept on ice. Dilution series of serine mutant peptides with concentrations ranging between 3.5 and 12 μM (S8E), 2.6 and 8 μM (S8Q), 1.7 and 10 μM (S26Q) or 6.4 and 13 μM (S26E) were prepared in low binding tubes (Genuine Axygen Quality, Microtubes, MCT-200-L-C). A 96-well PEG-coated polystyrene plate with a clear bottom (Corning 3881) was used to read the ThT fluorescence emitted from each mutant peptide sample. Each well was loaded with 80 μL of sample, and each mutant was studied in two different plates with triplicate samples for each concentration (*i.e.* six replicates of each condition). The plate was sealed with a plastic film (Corning 3095). The plate was placed in a Polarstar Omega plate reader (BMG Labtech, Offenburg, Germany) and incubated at 37 °C without shaking. The ThT fluorescence was measured every 200 s up to 24 h through the bottom of the plate, with the excitation and emission wavelengths at 440 and 480 nm, respectively. The half time (*t*_1/2_) was estimated by taking the values half-way in between the start and end baselines.

In the case of seeding experiments, fresh seeds were prepared from 10 μM monomer and fibrils were diluted into 30%, 10%, 3%, 1%, and 0.3% of the monomer concentration (4 μM). Four different seeding conditions were set up-self seeding of wt monomer (wtm) on wt seed (wts); self-seeding of mutant monomer (MUTm) on mutant seed (MUTs); cross-seeding of wtm on MUTs; cross-seeding of MUTm on wts. At least two different experiments with a minimum of three replicates was set up for each condition for both concentration dependence and seeding experiments.

### Cryo-TEM

Serine mutant monomers were prepared and incubated in the same way as for the aggregation kinetics at a concentration of 10 μM. All samples were collected when the fibril formation reached the plateau and immediately frozen. In a controlled environment vitrification system (CEVS), a 4 μL sample was loaded onto the lacey carbon-filmed copper TEM grid and blotted with a filter paper to absorb extra solution. The grid was then plunged into liquid ethane (−180 °C) to flash freeze all samples and stored in liquid nitrogen until imaged on the next day. Images were recorded at various magnifications by using the electron microscope JEM-2200FS.

### Data analysis

To extract the microscopic rate constants of primary nucleation, secondary nucleation and elongation, global analysis of aggregation kinetics of the serine phosphomimic mutants was performed using the online Amylofit platform.^69^ Two repeats of each mutant were uploaded, each with multiple concentrations in several replicates, normalized and fitted.

The differential equations describing the time evolution of aggregate number concentration, *P*(*t*), are6
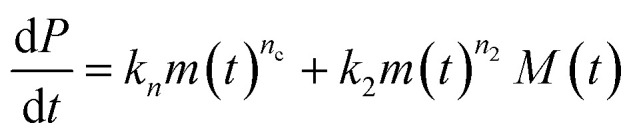
for a single step nucleation process and7
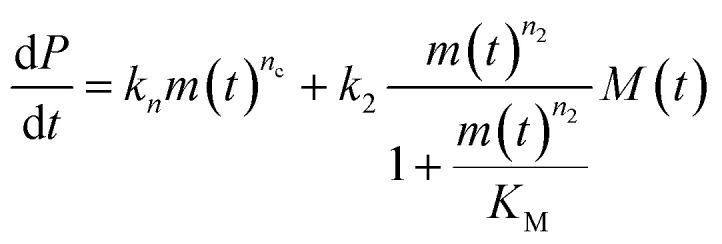
for a multi-step secondary nucleation process.

The time evolution of aggregate mass concentration, *M*(*t*), is8
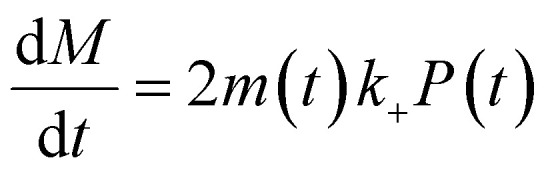
where *k*_*n*_, *k*_2_, *k*_+_ are the rate constants for primary nucleation, secondary nucleation and elongation respectively. *K*_M_ is the saturation constant for secondary nucleation (which is half saturated at (*K*_M_)^−*n*_2_^) and *n*_2_ and *n*_c_ are the monomer scalings (reaction orders) of primary and secondary nucleation, respectively. These models are used to fit the overall ThT fluorescence curves. Consequently, the rate constants are weighted averages over reactions involving any conformations that are present in the fibrillar or monomeric state. An approximate solution to eqn (1) and (2) is:9
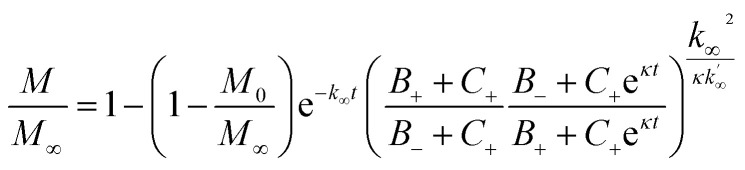
where the parameters are defined by10
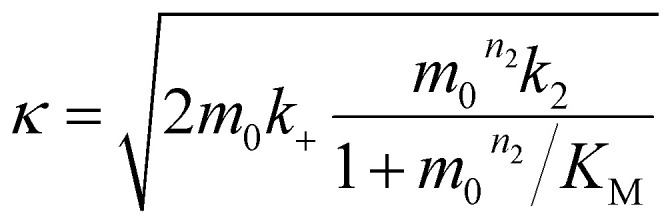
11
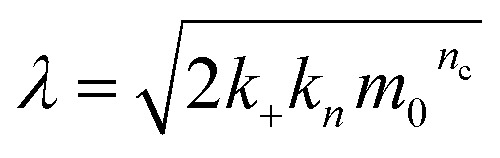
12
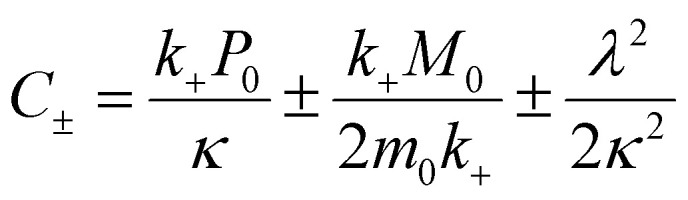

13 *k*_∞_ = 2*k*_+_*P*_∞_

14
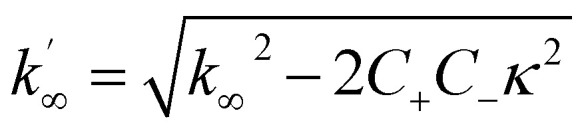
15
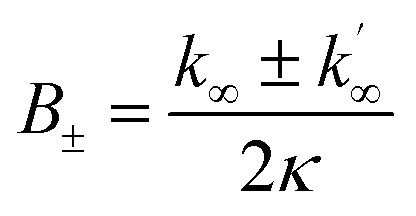
where *P*_0_ is the aggregate number at the start of the reaction, *P*_∞_ is the aggregate number at equilibrium (as described in ref. 69) that is, after reaction completion, *M*_0_ is the mass concentration of fibrils at the start of the reaction and *M*_∞_ is the mass concentration of fibrils at equilibrium. Reaction orders *n*_c_ = 2 and *n*_2_ = 2 were used for fitting of Aβ42 aggregation kinetics.

### Determination of elongation rate

The average size of aggregates, in numbers of monomers, is given approximately as16
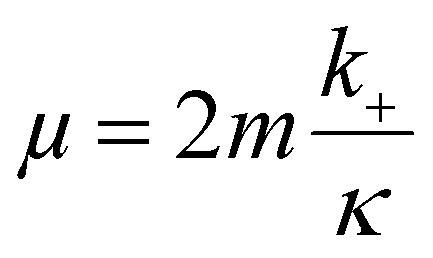


Thus, with a knowledge of the size of aggregates and the rate *κ*, the elongation rate constant *k*_+_ can be estimated. Using TEM measurements, which yield the average dimensions of the fibrils, and the assumption of a standard protein density of 1.3 kg L^−1^ and mass of Aβ42 of 4.5 kDa, we estimated the size of the aggregate in terms of the number of monomers. These values, along with the values of *κ* obtained from the fits of the kinetic data were then used to obtain the estimates of *k*_+_ shown in Fig. 4.

### Sample preparation for small- and wide-angle X-ray scattering

As described,^58^ the samples for the SAXS experiments were prepared by dissolving lyophilized powder of pure Aβ42 S26Q monomers in 20 mM sodium phosphate, 0.2 mM EDTA, 0.02% NaN_3_ at pH 8.0 to a final monomer concentration of 350 μM and a minimum volume of 200 μL. These S26Q monomer samples were incubated at 37 °C under quiescent conditions in low-binding tubes and analyzed by SAXS after 5 days in triplicate. One additional sample was analyzed after 3 months to investigate the fibril stability over time.

### Small- and wide-angle X-ray scattering

The experiments were performed by using a Saxslab Ganesha pinhole instrument, JJ X-Ray System Aps (JJ X-ray, Hoersholm, Denmark) with an X-ray microsource (Xenocs, Sassenage, France) and a two-dimensional 300 k Pilatus detector (Dectris Ltd, Baden-Daettwil, Switzerland). All measurements were performed in an air-evacuated space at a pressure below 1.6 mbar and at room temperature using Cu Kα radiation having a wavelength (*λ*) of 1.54 Å. Combined SAXS/WAXS experiments were performed in a *q* range from 0.004 to 2 Å^−1^. Samples were measured at three given sample-to-detector distances and the evolution of the scattering profile was monitored by data acquisition at different time points in order to detect possible changes in the sample over 24 h. No sedimentation or radiation damage effects were observed. The 2D-images from the Pilatus detector were azimuthally averaged after subtracting the dark counts. The background, recorded in a capillary with buffer at the same contrast, was subtracted from the acquired 1D scattering data, which are then plotted as *I*(*q*) *versus q*.

### Dot blot with A11

Each sample (10 μL) were spotted on two separate nitrocellulose membranes and let dry, followed by blocking for 60 min in gelatin blocking buffer from Sigma, and three washes with 50 mM Tris–HCl buffer, 150 mM NaCl, pH 7.6, 0.1% Tween-20 (wash buffer). The membrane was then incubated over night at 4 °C with primary antibody, anti-oligomer A11 polyclonal antibody (Invitrogen), 1 mg mL^−1^ stock at 1 : 1500 dilution in wash buffer plus 2.5% fish gelatin, followed by three washes with wash buffer. The membrane was then incubated with the secondary antibody – goat anti-rabbit IgG (H + L) poly-HRP (Invitrogen), 0.5 mg mL^−1^ stock at 1 : 2000 dilution in wash buffer plus 2.5% fish gelatin for 60 min, followed by three washes with wash buffer. The membrane was developed using chemiluminescent substrate (Pierce™ ECL Western Blotting Substrate; Thermo Scientific™) for 1 min and imaged using autoexposure in Chemidoc imaging system (BioRad).

## Conclusions

The results of this study rationalize the effect of phosphorylation of Aβ, yielding insights into the changes in the mechanism of aggregation due to size and charge modification at the two serine residue positions in the secondary nucleation process. Our data show a clear position-dependence in the role of phosphorylation and in particular the identity of the residue at position 26 in Aβ42 plays a distinct role in the nucleation at the fibril surface and in the stabilization of a specific fibril structure. The propensity to catalyse nucleation of wt monomers is diminished for fibrils with a glutamate or glutamine rather than serine residue at this position. This is likely related to the formation of variant fibril structures, which are unstable with a serine residue at position 26 as in Aβ42 wt, while the Aβ42 wt structure is less stable than the variant structures when a bulkier glutamine or glutamate residue occupies position 26. The smaller cross section dimensions for S26Q compared to wt fibrils may reflect tighter packing of the two filaments, a different rotation of the two filaments or a change in the fibril core packing, *i.e.* the monomer fold in the fibrils. Cross-seeding thus seems to be possible only between peptides that can form fibrils of the same or highly similar structure.

The results for our phosphomimic mutants may be extrapolated to phosphorylated wt. We thus predict that phosphorylation of Ser8 may reduce the nucleation rate, but this modification will be compatible with the fibril structure of non-phosphorylated wt and will allow cross seeding between phosphorylated and regular peptide. We also predict that phosphorylation of Ser26 will have a more significant retarding effect and will lead to the formation of fibrils with a different structure than non-phosphorylated Aβ42 and the position 26 phosphorylated peptide fails to form joint fibrils with the wt.

## Data availability

All data in this manuscript will be deposited in our github account and be made public upon acceptance and publication of this work.

## Author contributions

AM, SL designed study. KS, VL, SL performed experiments. KS, GM, VL, UO, TPJK, SL analyzed data. KS, KB, BF, SL provided the materials. KS, SL wrote the manuscript with input from all co-authors.

## Conflicts of interest

The authors have no conflicts of interest with the content of this work.

## Supplementary Material

SC-OLF-D3SC06343G-s001
