# Supplementary material for: Serine phosphorylation mimics of Aβ form distinct, non-cross-seeding fibril morphs
Source: Chem Sci. 2024 Oct 11;15(45):19142–59. doi: 10.1039/d3sc06343g (PMC11529392; doi:10.1039/d3sc06343g)
Supplement: SC-015-D3SC06343G-s001 [file SC-015-D3SC06343G-s001.pdf]

Supplementary Information (SI) for Chemical Science.
This journal is © The Royal Society of Chemistry 2024

Serine phosphorylation mimics of Aβ form distinct, non-cross-seeding fibril morphs

Kalyani Sanagavarapu[a*], Georg Meisl[b], Veronica Lattanzi[a,d], Katja Bernfur[a], Birgitta Frohm[a], Ulf Olsson[d], Tuomas PJ Knowles[b,c], Anders Malmendal[a,e], Sara Linse[a*]

a) Lund University, Biochemistry and Structural Biology, Department of Chemistry, Lund, Sweden.
b) University of Cambridge, Yusuf Hamied Chemistry Department, Lensfield Road, Cambridge, UK.
c) University of Cambridge, Cavendish Laboratory, Department of Physics, JJ Thomson Avenue, Cambridge UK.
d) Lund University, Physical Chemistry, Department of Chemistry, Lund, Sweden.
e) Roskilde University, Department of Science and Environment, Roskilde, Denmark.
* Corresponding authors

Supplementary information

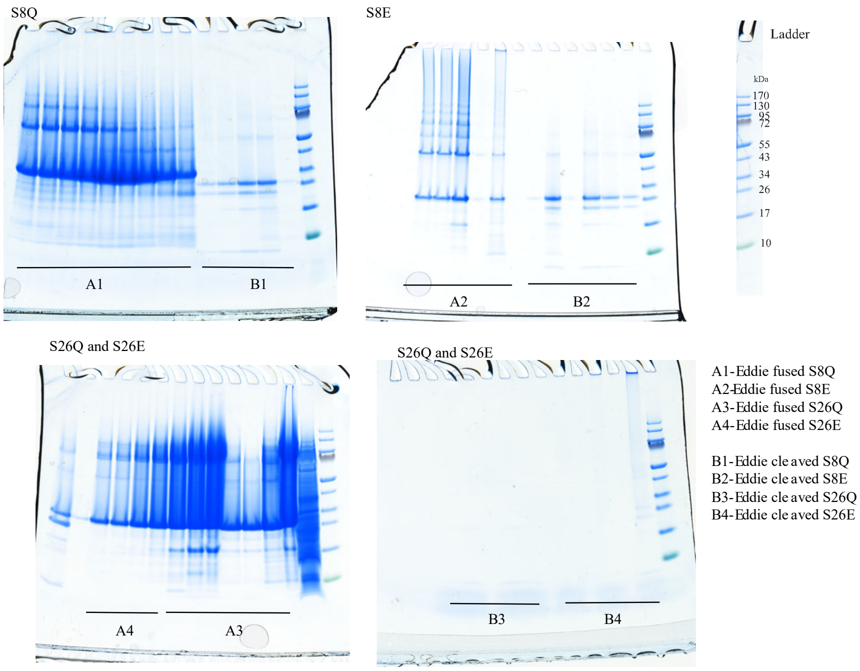

Figure S1. Purification of S8Q, S8E, S26Q an S26E mutants. Samples labelled A are fused EDDIE constructs, samples labelled B are EDDIE cleaved peptides after 72 h. The B3 and B4 samples are purified by anion exchange chromatography after 72 h of EDDIE cleavage.

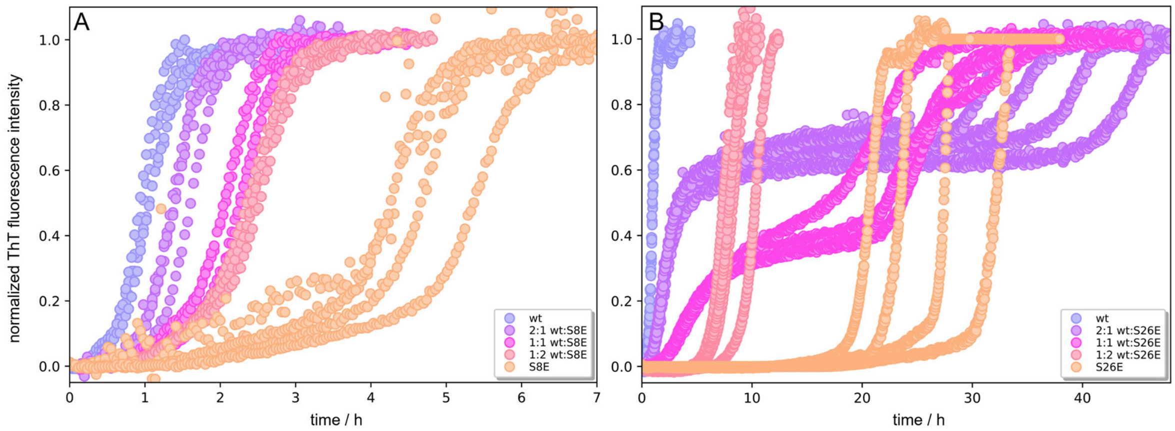

Figure S2. Co-aggregation data for monomer mixtures with constant total concentration. A. Co-aggregation data for samples of WT and S8E at a total concentration of 3.2 μM at five different wt:S8E ratios - 1:0, 2:1, 1:1, 1:2, 0:1 – with the color codes shown in the inset. **B.** Co-aggregation data for samples of WT and S26E at a total concentration of 4 μM at five different wt:S26E ratios - 1:0, 2:1, 1:1, 1:2, 0:1 – with the color codes shown in the inset. All experiments were performed in quadruplicate and all traces are shown.

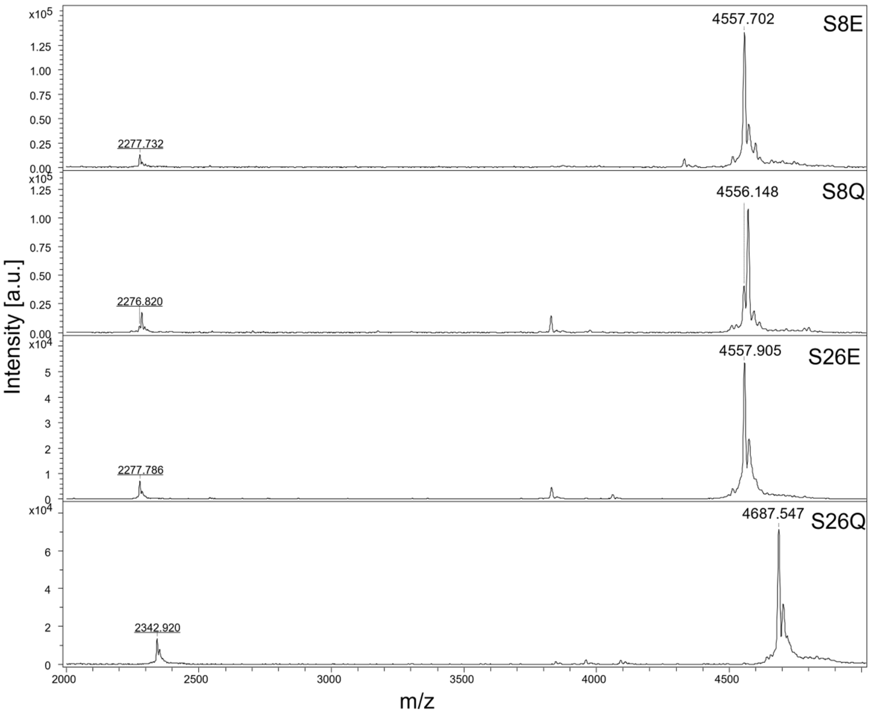

Theoretical average masses:

Aβ1-42 S8E: 4557.08

Aβ1-42 S8Q: 4556.10

Aβ1-42 S26E: 4557.08

Aβ(M1-42) S26Q: 4687.30

Figure S3. MALDI mass spectra obtained on monomer samples in linear mode with external calibration for the four Aβ42 mutants with their theoretical average masses given to the right.

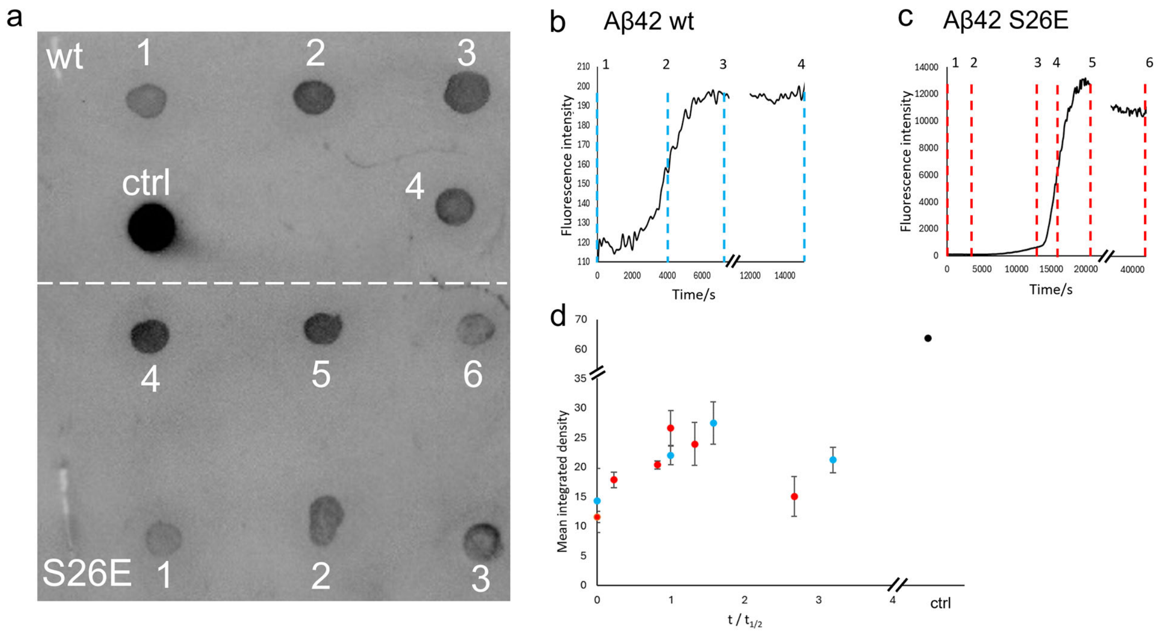

Figure S4. A11 dot blot. Dot blot of Aβ42 wt and S26E, both at a starting monomer concentration of 15 µM using A11 primary antibody. All samples were incubated in 20 mM sodium phosphate buffer, 0.2 mM EDTA, 0.02% NaN_3 pH 8.0, in a plate reader. Some wells contained no ThT and some contained 6 µM ThT. Samples for the dot blot (10 µl) were collected from wells without ThT. Samples 1 of Aβ42 wt and S26E were spotted right after eluting from SEC. Samples 2-6 were collected along the reaction time-course as guided by the fluorescence signal from the wells sample with ThT. The control spot, ctrl, is 10 µL of primary antibody (A11) at 10 µg/mL. **b,c.** ThT fluorescence intensity (a.u. = arbitrary units) versus time for Aβ42 wt and S26E, with blue (wt) and red (S26E) lines indicating the time points of samples collected for spotting. **d.** Integrated density values of Aβ42 wt (blue) and S26E (red) samples and the control (ctrl) as analyzed by ImageJ. The mean and standard deviation is over four readings from two separate blots.
